# Qingxin Kaiqiao Fang Inhibits A*β*_25-35_-Induced Apoptosis in Primary Cultured Rat Hippocampal Neuronal Cells via the p38 MAPK Pathway: An Experimental Validation and Network Pharmacology Study

**DOI:** 10.1155/2020/9058135

**Published:** 2020-08-03

**Authors:** Tian-Qi Wang, Xiao-Xiao Lai, Lu-Ting Xu, Yan Shen, Jian-Wei Lin, Shi-Yu Gao, Hai-Yan Hu

**Affiliations:** ^1^The Second Affiliated Hospital and Yuying Children's Hospital of Wenzhou Medical University, 109 Xue Yuan Xi Road, Lu Cheng District, Wenzhou 325000, China; ^2^The Second Clinical College, Wenzhou Medical University, Wenzhou 325003, China

## Abstract

Qingxin kaiqiao fang (QKF), a traditional Chinese medicine compound, has been applied to treat Alzheimer's disease (AD) for many years and has exhibited remarkable effects. However, the underlying mechanism is still not explicit. The current study aims to investigate whether QKF exerts an antiapoptotic role through the p38 MAPK pathway in the course of AD. Network pharmacology analysis was applied to study the effective components, possible therapeutic targets, and AD-related pathway of QKF. Further, the AD cell model was established using amyloid-beta (A*β*)_25-35_ peptide and primary hippocampal neuronal cells extracted from newborn Sprague-Dawley rats. Microtubule-associated protein-2 (MAP-2) imaging was used to detect the morphology of hippocampal neurons. Western blot (WB) analysis was applied to detect the protein expression levels of p38 MAPK, p-p38 MAPK, Bcl-2, Bax, caspase-3, and cleaved caspase-3. Cell viability and apoptosis were determined using cell counting kit-8 (CCK-8) and terminal deoxynucleotidyl transferase-mediated dUTP nick end-labeling (TUNEL) assays, respectively. SB203580 and U46619 were used to detect changes in cell morphology, cell viability, and apoptosis upon inhibiting or activating p38 MAPK. Our present work showed that QKF protects hippocampal neuronal morphology, enhances cell viability, and reduces the number of TUNEL-positive cells. In addition, our results showed that QKF increased the expression levels of antiapoptotic proteins and decreased the expression of proapoptotic proteins. QKF at 25 mg·mL^−1^ best inhibited neuronal apoptosis among the three doses of QKF by suppressing p38 MAPK activity. Collectively, QKF plays an antiapoptotic role via the p38 MAPK pathway.

## 1. Introduction

As a serious neurodegenerative disease in aged individuals, Alzheimer's disease (AD) is characterized by the formation of intracellular neurofibrillary tangles, extracellular deposits of senile plaques (SP), and the loss of neurons [1]. It is generally accepted that the apoptosis-induced death of a large number of neurons is a common feature in the brains of patients with AD [2]. This programmed cell death can be induced by multiple factors, one of which is the abnormal aggregation and deposition of amyloid-beta (A*β*) fragments, the main component of SP. Among the A*β* fragments studied to date, A*β*_25-35_ is the shortest, but it retains the toxicity of the full-length peptide and exhibits a significant level of molecular aggregation [3, 4]. Evidence suggests that the excessive deposition of A*β* in the hippocampus leads to changes in nerve function and eventually cognitive dysfunction, as the hippocampus is one of the most delicate areas in the brain and primarily responsible for learning and memory [5]. Moreover, some studies have shown that hippocampal neuronal damage induced by A*β* can be mediated by the regulation of p38 mitogen-activated protein kinase (p38 MAPK) [6, 7]. A member of the MAPK family, p38 MAPK, is closely related to the regulation of cell proliferation, differentiation, survival, and death. Recent studies have found that its phosphorylation is markedly increased in both the hippocampi of AD rats and in cultured neurons [8, 9]. In addition, p38 MAPK activation leads to programmed neuronal cell death primarily through alterations in the expression of proteins involved in apoptosis, including caspase-3, the antiapoptotic protein regulator Bcl2, and the proapoptotic protein regulator Bax [10]. In view of the role of p38 MAPK in apoptosis, SB203580, a direct inhibitor of p38 MAPK, and U46619, an agonist of p38 MAPK, are currently applied by many researchers to assess whether the antiapoptotic role of p38 MAPK is mediated by its suppression [11, 12]. Qingxin kaiqiao fang (QKF), which is based on Fumanjian, a notable traditional Chinese medicine compound from Jingyue Quanshu that was first described by Zhang Jingyue during the Ming Dynasty, is made mainly from Radix Rehmanniae (Sheng Di Huang), Radix Paeoniae Alba (Bai Shao), Radix Ophiopogonis (Mai dong), Cortex Moutan Radicis (Mu dan pi), *Poria cocos* (Fu Ling), Herba Dendrobii Rhizoma (Shi Hu), Rhizoma Acori Tatarinowii (Shi Chang Pu), Rhizoma Anemarrhenae (Zhi mu), Sophorae Flavescentis (Ku Shen), and Pericarpium Citri Reticulatae (Chenpi). A network pharmacology analysis, which integrates high-throughput data integration, database retrieval, data mining, target prediction, laboratory simulations, and other research methods [13], was applied in this study to reveal the complex mechanism of QKF in the treatment of AD.

## 2. Materials and Methods

### 2.1. Active Ingredients Screening and Targets Identification

The BATMAN-TCM database (a bioinformatics analysis tool for determining the molecular mechanism of TCM) (http://bionet.ncpsb.org/batman-tcm/) was used to search for the compounds contained in QKF. Further, we predicted the potential targets of the above drugs based on the BATMAN-TCM database and set a target score≥20 as the threshold for targets. Afterward, a “QKF-compound-target” network was constructed and analyzed using Cytoscape 3.7.2 software. AD-related targets were retrieved from the following five databases, namely, Online Mendelian Inheritance in Man (OMIM; https://www.omim.org/), PharmGKB (http://www.pharmgkc.org/), GeneCards (http://www.genecards.org/), GAD (https://geneticassociationdb.nih.gov/), and KEGG (http://www.kegg.jp/) databases.

### 2.2. Network Establishment

The drug-related targets and disease targets were standardized by using the UniProt database. The String database (https://string-db.rog/) was used to analyze protein-protein interactions (PPIs). Afterward, the network analyzer function of Cytoscape 3.7.2 software was used to analyze the network parameters. The topological analysis was used to identify the key genes for which the network degrees and betweenness were greater than the mean values.

### 2.3. Gene Pathway and Functional Analysis

The core target genes were identified using the DAVID database (http://david.nifcrf.gov/). The signaling pathways and biological processes of QKF relevant to the treatment of AD were analyzed by gene ontology (GO) enrichment analysis (http://www.geneontology.org) and the Kyoto Encyclopedia of Genes and Genomes (KEGG) pathway enrichment analysis (http://www.kegg.jp/) (*P* ≤ 0.01).

### 2.4. Main Reagents

Poly-L-lysine (PLL) (10x) was purchased from Beijing Solarbio Science and Technology Co., Ltd. (Beijing, China). DMEM/F12 (1 : 1), Neurobasal Plus Medium, B27 Plus Supplement (50x), HBSS (1x), FBS, and 0.25% trypsin were obtained from Gibco (Grand Island, New York, USA). The A*β*_25-35_ fragment and dimethyl sulfoxide (DMSO) were purchased from Sigma (St. Louis, MO, USA). Donepezil hydrochloride, SB203580, and U46619 were obtained from Santa Cruz Biotechnology Inc. (Delaware, USA). L-Glutamine was obtained from Thermo Fisher (Massachusetts, USA). The rabbit polyclonal antibodies against MAP2 were purchased from Proteintech Group, Inc. (Chicago, USA). Rabbit monoclonal antibodies against p38 MAPK, p-p38 MAPK, caspase-3, cleaved caspase-3, and Bax were purchased from Cell Signaling Technology (Beverly, MA, USA). The primary antibody against Bcl-2 was purchased from Abcam (Cambridge, UK). Horseradish peroxidase- (HRP-) conjugated IgG secondary antibody was purchased from Beijing Bioss Biotechnology Co., Ltd. (Beijing, China), and the BCA protein assay kit was obtained from Beyotime Biotechnological Technology Co., Ltd. (Shanghai, China).

### 2.5. Preparation of QKF and A*β*_25-35_

QKF is composed of 10 herbs: Radix Rehmanniae (Sheng Di Huang), Radix Paeoniae Alba (Bai Shao), Radix Ophiopogonis (Mai dong), Cortex Moutan Radicis (Mu dan pi), *Poria cocos* (Fu Ling), Herba Dendrobii Rhizoma (Shi Hu), Rhizoma Acori Tatarinowii (Shi Chang Pu), Rhizoma Anemarrhenae (Zhi mu), Sophorae Flavescentis (Ku Shen), and Pericarpium Citri Reticulatae (Chenpi), all of which are recorded in the Chinese Pharmacopoeia, as being present in QKF at a ratio of 2 : 2: 2 : 2: 2 : 2: 2 : 1.5 : 1.5 : 1 on a dry-weight basis, respectively. All herbs were provided by the Second Affiliated Hospital of Wenzhou Medical University and verified by the Department of Chinese Materia Medical of Wenzhou Medical University. To make the 1 g·mL^−1^ stock solution, the raw herbs were decocted with 10 times the volume of distilled water, extracted twice, filtered, and concentrated, and the drug stocks were then sterilized in an autoclave (Panasonic Co., Ltd. Tokyo, Japan; MLS-3751L) and stored at 4°C until use. The A*β*_25-35_ peptide was dissolved in DMSO at a concentration of 100 *μ*mol L^−1^ and then placed in a 37°C thermostat water bath three days prior to use. Prior to use, it was diluted to 10 *μ*mol L^−1^ with a maintenance medium.

### 2.6. Primary Hippocampal Neuron Cultures

One-day-old Sprague-Dawley (5-6 g) rats were purchased from Beijing Vital River Laboratory Animal Technology Co., Ltd. (certification number SCXK 2012-0001; Beijing). The rats were sterilized using 75% ethanol and then euthanized. The skin and skull were cut with a pair of sterile scissors. The brain was removed from the skull with a pair of tweezers and placed in a small Petri dish with a small amount of HBSS. The cerebral hemispheres were peeled back, and the hippocampus was removed and placed in a Petri dish with the proper amount of HBSS. After all hippocampi were isolated, they were cut into 1 mm sections with a pair of ophthalmic scissors and washed with PBS, and then 3 ml of 0.125% trypsin was added to the sections which were incubated for 15 min at 37°C. Fifteen minutes later, the same amount of DMEM/F12, containing 10% FBS and 1% penicillin-streptomycin solution, was added to terminate cell digestion, and the solution was gently triturated into a cell suspension with a sterile pipette. The cell suspension was then filtered with a 200-mesh copper sieve, and the filtered cell suspension underwent centrifugation (4°C; 500 r) for 2 min, after which the supernatant was retained. The retained suspension again underwent centrifugation (4°C; 1000 r) for 5 min, and the supernatant was discarded, following which fresh culture medium was added. Finally, the cells were placed in an incubator with 5% CO_2_ and incubated at 37°C for 1 h to induce differential adhesion. Following cell counting using a blood cell counting chamber, the cells were seeded in a 6-well plate at a density of 2 × 10^6^/well. The culture medium was replaced with maintenance medium composed of Neurobasal Plus Medium, B27 Plus, L-glutamine, and penicillin-streptomycin solution four hours later. Thereafter, half of the maintenance medium was replaced with fresh medium every two days. The abovementioned animal experiments were conducted in accordance with the ethical requirements approved by the Chinese Association of Accreditation of Laboratory Animal Care.

### 2.7. Cell Grouping

After 7 days of culture, the neurons were randomly grouped as follows: control group; model group, which was then treated with 10 *μ*mol·L^−1^ A*β*_25-35_ alone on the 8^th^ day of culture; 25 mg mL^−1^ QKF group; 12.5 mg mL^−1^ QKF group; 6.25 mg mL^−1^ QKF group; donepezil, which was used as a positive control group and treated with 10 *μ*mol·L^−1^ donepezil hydrochloride; 25 mg mL^−1^ QKF + SB203580 group, which was treated with 10 *μ*mol L^−1^ SB203580 and 25 mg mL^−1^ QKF; and 25 mg mL^−1^ QKF + *U*46619 group, which was treated with 10 *μ*mol·L^−1^ U46619 and 25 mg mL^−1^ QKF. After 24 h of drug treatment, A*ββ*_25-35_ (at a final concentration of 10 *μ*mol L^−1^) was added to all groups (except for the control group) for 24 h to establish the AD cell model. Meanwhile, the control group was incubated with the same volume of culture medium for the same length of time.

### 2.8. Cell Observation

Cell morphology was observed under a Nikon inverted fluorescent microscope (Nikon Corp., Tokyo, Japan) at 4 h and 1, 3, 5, and 7 days. After 9 days of culture, the neurons were washed with PBS three times (5 min each time), fixed with 4% paraformaldehyde for 20 min at room temperature, washed with PBS another three times, permeated with 0.2% Triton X-100 at room temperature, washed with PBS a further three times, blocked with 5% BSA, and finally washed with PBS three more times. The neurons were cultured with anti-MAP2 polyclonal antibody (1 : 200) in a wet box at 4˚C for 24 h, washed with PBS three times, incubated with anti-rabbit IgG (*H* + *L*) at 37°C for 1 h in the dark, and washed with PBS three times. The neurons were incubated with DAPI for 10 min at room temperature, treated with antifluorescence quenching and sealing tablets, and washed with PBS three times (5 min each time). Images of neurons and nuclei were captured under a high-power microscope.

### 2.9. Western Blot Analysis

The total protein was extracted with lysis buffer, and the total protein concentration was determined using the bicinchoninic acid assay. Proteins were separated by 10% PAGE and subsequently transferred onto polyvinylidene difluoride (PVDF) membranes (Roche Applied Science, Indianapolis, IN, USA). The membranes were blocked for 2 h in skim milk blocking buffer and washed 3 times with TBS/0.1% Tween 20 before incubation with primary antibodies (1 : 1000) against p38 MAPK, p-p38 MAPK, Bcl2, Bax, caspase-3, cleaved caspase-3, and *β*-tubulin overnight at 4°C. The membranes were then incubated with HRP-conjugated goat anti-rabbit secondary antibody as appropriate for 2 h at room temperature. After being washed three times with TBST, the immunoreactive proteins were visualized using an ECL Plus reagent kit. Finally, the density of each band was quantified using AlphaEaseFC, and *β*-tubulin served as the internal control.

### 2.10. Cell Viability Assay

Cell viability was assessed by cell counting kit-8 (CCK-8) assay (Dojindo Laboratories, Tokyo, Japan) according to the manufacturer's instructions. In short, hippocampal neurons were plated directly in 96-well plates at a density of 1 × 10^4^ cells/well and grouped into five wells for each group. After treatment with the corresponding drugs for 24 h, the cells were exposed to A*β*_25-35_ (10 *μ*mol L^−1^) for an additional 24 h and then cultured in maintenance medium (100 *μ*L) and CCK-8 reagent (10 *μ*L) for 4 h at 37°C. Finally, cell viability was determined by reading the optical density (OD) at a wavelength of 450 nm using an enzyme-labeled instrument (Infinite 200Pro, Tecan, Switzerland).

### 2.11. TUNEL Staining

TUNEL staining was performed with an In Situ Cell Death Detection Kit (Roche, Mannheim, Germany), according to the manufacturer's instructions. Neurons at day 9 were washed with PBS three times (5 min each time), fixed with 4% paraformaldehyde for 20 min at room temperature, washed with PBS another three times, permeated with 0.1% Triton X-100 in 0.1% sodium citrate for 2 min on ice, and washed with PBS a further three times. The cells were then incubated with 50 *μ*L of TUNEL reaction mixture in a humidified atmosphere for 1 h at 37°C in the dark, rinsed three times with PBS, subjected to fluorescent counterstaining with DAPI for 10 min at room temperature, washed with PBS three times, and finally treated with an antifluorescence quenching sealing tablet. The ratio of TUNEL-positive cells to the total number of cells was determined under a light microscope (Leica DM 3000) and used to calculate the proportion of apoptotic.

### 2.12. Statistical Analysis

SPSS 22.0 statistical software (IBM Corporation, Armonk, NY, USA) was used to analyze the data. The measurement data are expressed as the mean ± standard deviation. The data from different groups were compared using the independent-samples *t*-test and one-way ANOVA. Pairwise comparisons of homogeneous data were performed using the least significant difference (LSD) test. *P* < 0.05 indicated statistical significance for all analyses.

## 3. Results

### 3.1. Identification of Active Ingredients and Target Selection

**Figure 1:**
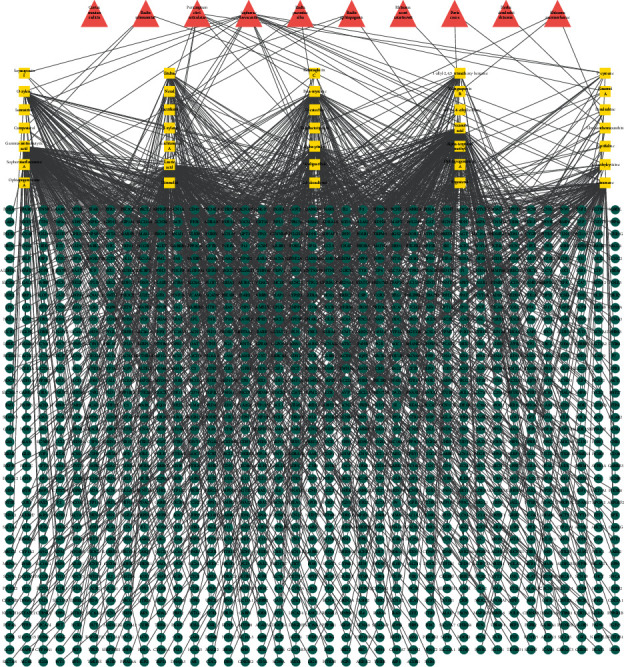
Compound-target network diagram. The red triangle represents herbs in Qingxin kaiqiao fang, yellow diamond indicates candidate compounds, and the green circle indicates predicted targets.

A total of 295 active compounds were identified in the ten herbs contained in QKF. The 9 active compounds in Radix Rehmanniae were identified. Thirty-five active compounds were identified in Radix Paeoniae Alba. Twenty-two active compounds were identified from Radix Ophiopogonis. Eighteen active compounds were identified in Cortex Moutan Radicis. Twenty-one active compounds were identified in *Poria cocos*. Twelve active compounds were identified in Herba Dendrobii Rhizoma. Fourteen active compounds were identified in Rhizoma Acori Tatarinowii. Thirty-two active compounds were identified in Rhizoma Anemarrhenae. A total of 116 active compounds were identified from Sophorae Flavescentis. Thirty-five active compounds were identified in Pericarpium Citri Reticulatae (Supplementary Tables 1 and 2). A total of 7433 target proteins were identified in QKF. Topological analysis of the protein interaction network nodes revealed a total of 1368 target proteins when the duplicates were removed. We constructed an ingredient-target network (Supplementary Table 3) (Figure 1). For disease target identification, we identified the AD-related targets in the OMIM, GeneCards, PharmGKB, GAD, and KEGG databases. We retrieved 1689 target genes corresponding to disease targets and identified 1218 target genes by screening (Supplementary Table 4).

### 3.2. PPI Network Construction and Functional Enrichment Analysis of QKF

**Figure 2:**
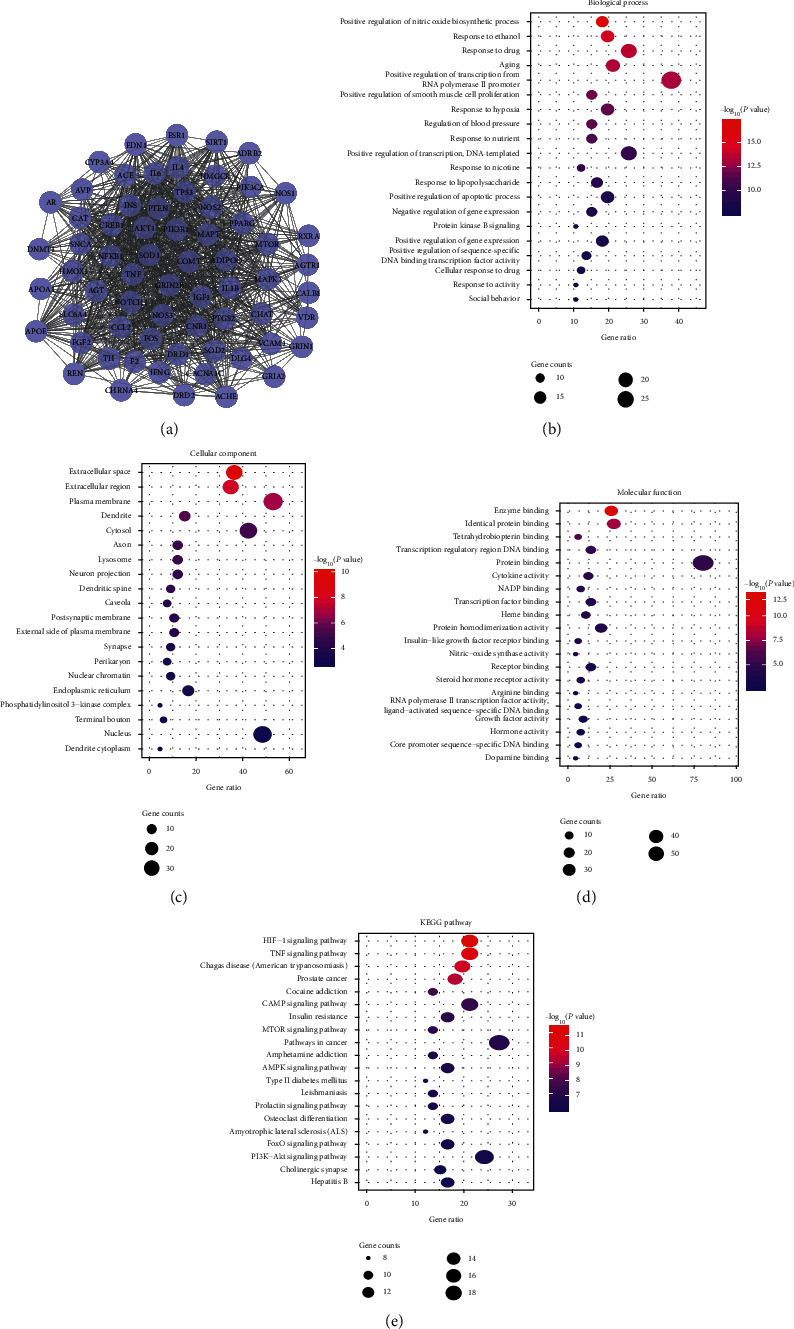
Network construction and enrichment analysis. (a) The blue circle represents the key target of Qingxin kaiqiao fang in the treatment of Alzheimer's disease; the edge represents the interaction between the targets predicted by String database, and the thicker the line is, the higher the confidence level is. (b) Biological process in GO enrichment analysis. (c) Cellular component in GO enrichment analysis. (d) Molecular function in GO enrichment analysis. (e) KEGG pathway enrichment analysis. GO, gene ontology; KEGG, Kyoto Encyclopedia of Genes and Genomes.

There is a certain degree of signaling transduction between different signal pathways and targets. Therefore, the mechanism of action between targets can be analyzed by determining the interactions between proteins. We used String 11.0 to determine the PPIs among the 1368 targets of QKF and the 1218 targets related to AD, which include 284 nodes and 4183 edges. According to the established screening rules, 66 targets were identified as key targets related to the QKF treatment of AD (Supplementary Table 5) (Figure 2(a)). GO enrichment analysis revealed 58 entries related to AD (Supplementary Tables 6, 7, and 8) (Figures 2(b)–2(d)). KEGG enrichment analysis identified 22 pathways (*P* < 0.01) (Supplementary Table 9) (Figure 2(e)). The results indicated that apoptosis may be the main way where QKF exerts its effects during the treatment of AD. Therefore, we designed an in vitro experiment to verify our hypothesis.

### 3.3. QKF Protected Hippocampal Neuron Morphology

**Figure 3:**
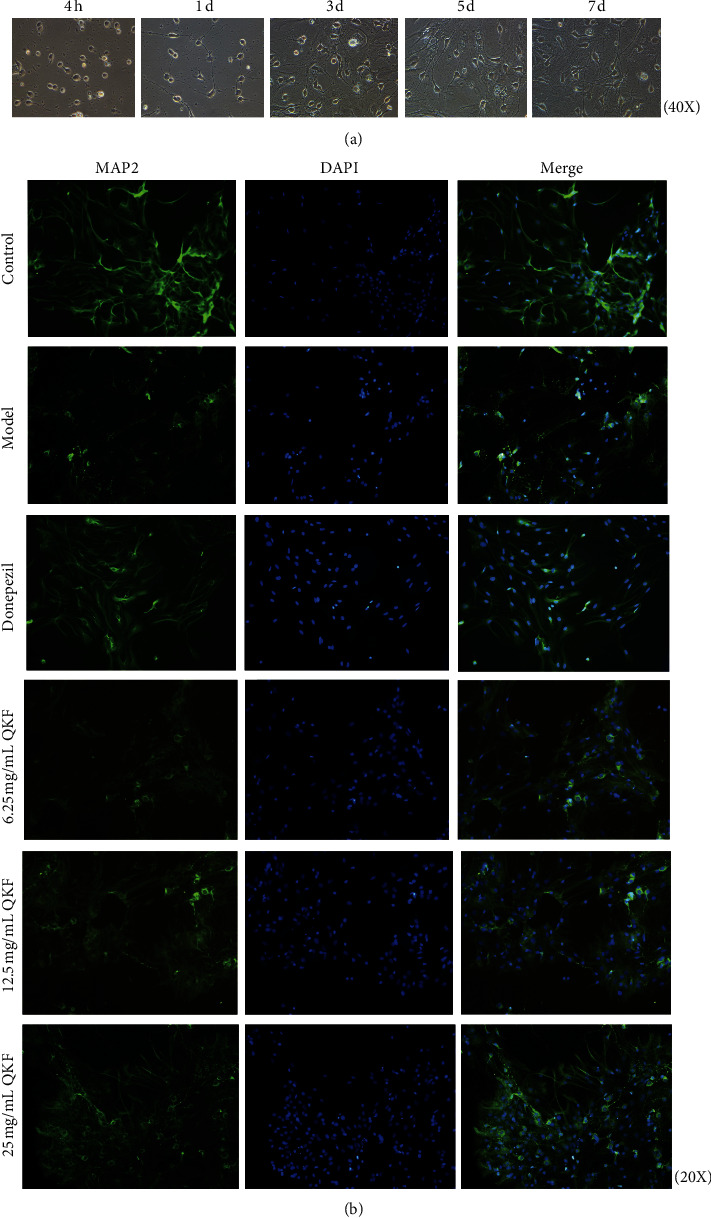
Qingxin kaiqiao fang reduces the damage of A*ββ*_25-35_ to cell morphology. (a) Morphology characteristic of hippocampal neuronal cells at different stages (4 h, 1 day, 3 days, 5 days, and 7 days, respectively) (original magnification, ×400). (b) Immunofluorescence images of microtubule-associated protein-2 (green) and nucleus (blue) (original magnification, ×200).

The morphology and growth of rat hippocampal neurons were observed under Nikon inverted fluorescence microscope at 4 h and 1, 3, 5, and 7 days (Figure 3(a)). Hippocampal neurons were resuspended just after their inoculation. Four hours later, most of the cells were adherent and presented a rounded shape that was surrounded by a halo; a few cells also showed protrusions. At day 1, the neurons were completely adherent; grew well with protrusions of different lengths; and showed a spindle-like, triangular, or irregular morphology; however, no clear connections between neurons were established. After 3 days of culture, the number of cell protrusions was increased, the cells were obviously thickened and elongated in shape, and connections between neurons began to form. After 5 days of culture, the neurons were plump with a surrounding halo and had become more connected. After 7 days of culture, the cell body was enlarged and plump, there was an obvious halo around the cells, and the synapse had become longer and thicker. The neurons had moved closer to each other and began to form cell populations, and the cell protrusions had formed a dense nerve fiber network. We next examined the neuronal cell morphology in each group following different treatments using an immunofluorescence microscope to image microtubule-associated protein-2 (MAP-2), which was predominantly localized within cell bodies and dendrites. The neurons in the control group were plump and dense, and their cell protrusions formed a dense and elaborate nerve fiber network. The neurons in the group treated with A*β*_25-35_ alone showed a smaller cell body with thinner and more damaged dendrites than those in the control group and sometimes presented a fragmented or beaded appearance, and the number of connections between neurons was decreased. However, after treatment with donepezil or QKF, the cell body became plumper, the dendrites became thicker, and the junctions between cells were tighter. The 25 mg mL^−1^ QKF group showed an improved effect compared to that in the QKF groups at the other tested doses (Figure 3(b)).

### 3.4. QKF Alleviated Neuronal Apoptosis Induced by A*β*_25-35_

**Figure 4:**
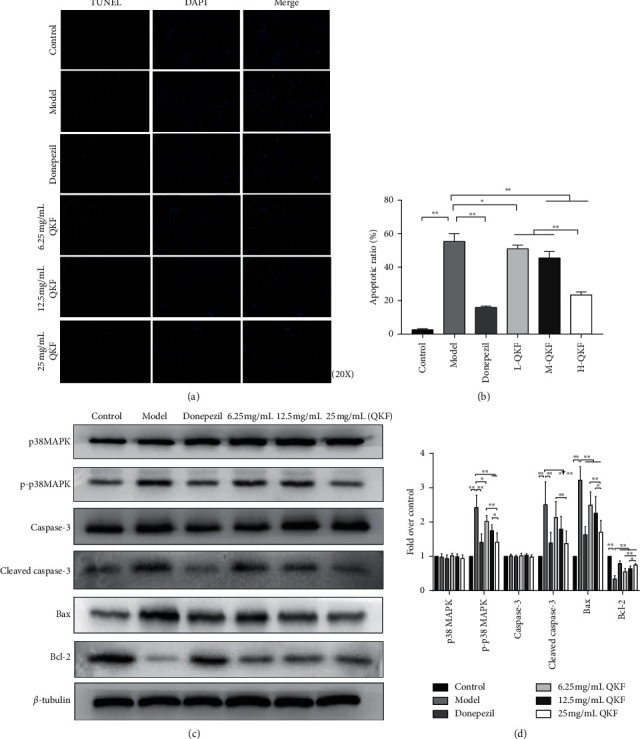
Qingxin kaiqiao fang reduces hippocampal neuronal apoptosis. (a) TUNEL staining of hippocampal neurons in each group (original magnification, ×200). (b) Statistical analysis results of TUNEL staining. (c) The gray value of p38 MAPK, p-p38 MAPK, caspase-3, cleaved caspase-3, Bax, and Bcl-2 in each group. (d) ODs indicative of p38 MAPK, p-p38 MAPK, caspase-3, cleaved caspase-3, Bax, and Bcl-2 protein expression. Data are expressed as mean ± SD (*n* = 5), significant differences between the two groups are indicated as ^*∗*^*P* < 0.05 and ^*∗∗*^*P* < 0.01. QKF, Qingxin kaiqiao fang.

A TUNEL assay was performed to examine apoptosis in hippocampal neurons. TUNEL-positive cells in the selected visual field were counted by the positive cell counting method, and the final values are presented as the ratio of the number of TUNEL-positive cells to the number of total cells. As shown in Figures 4(a) and 4(b), the apoptotic rate of the hippocampal neurons in the model group was visibly increased compared with that in the control group (*P* < 0.01). In comparison with that in the model group, treatment with QKF and donepezil reduced the apoptotic rate (*P* < 0.05, *P* < 0.01). The 25 mg mL^−1^ QKF group showed a greater decrease than the other two QKF groups that were tested (*P* < 0.01).

The expression levels of apoptosis-related proteins in rat hippocampal neurons were measured by Western blot (WB) analysis (Figures 4(c), 4(d)). There were no evident differences in the total p38 MAPK or caspase-3 protein expression levels among the six groups (*P* > 0.05). WB analysis showed significantly lower Bcl-2 protein levels in the model group compared with those in the control group (*P* < 0.01). The Bcl-2 protein expression level in the QKF and donepezil groups was increased compared to that in the model group (*P* < 0.01). QKF at 25 mg mL^−1^ increased the expression of Bcl-2 to the greatest extent among the three doses of QKF examined (*P* < 0.05, *P* < 0.01). The expression levels of p-p38 MAPK, cleaved caspase-3, and Bax were increased in the model group compared with those in the control, donepezil, and 25 mg mL^−1^ QKF groups (*P* < 0.01). Compared with those in the model group, the expression levels of Bax were decreased in the 6.25 mg mL^−1^ QKF and 12.5 mg mL^−1^ QKF groups (*P* < 0.01), and the expression levels of p-p38 MAPK were decreased in the 6.25 mg mL^−1^ QKF (*P* < 0.05) and 12.5 mg·mL^−1^ groups (*P* < 0.01). The 12.5 mg mL^−1^ QKF group exhibited downregulated expression levels of cleaved caspase-3 (*P* < 0.05), while the 6.25 mg mL^−1^ QKF group did not show an evident difference in the expression of cleaved caspase-3 compared with the model group (*P* > 0.05). Moreover, the altered protein expression levels in both the 25 mg mL^−1^ QKF and donepezil groups showed a consistent trend, and there were no clear differences in the effects between these two groups (*P* > 0.05).

### 3.5. QKF Increased Cell Viability

**Figure 5:**
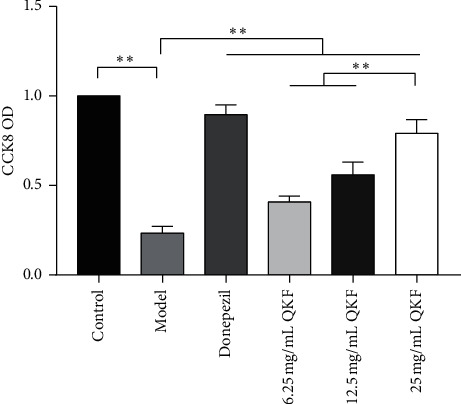
CCK8 assay demonstrates that Qingxin kaiqiao fang promotes the cell viability of injured hippocampal neurons. Data are expressed as mean ± SD (*n* = 5); significant differences between the two groups are indicated as ^*∗∗*^*P* < 0.01. QKF, Qingxin kaiqiao fang.

A CCK-8 assay was used to test hippocampal neuron viability. As shown in Figure 5, there was a sharp decline in hippocampal neuron viability after 24 h of treatment with A*β*_25-35_ alone compared with that in the control group (*P* < 0.01). In comparison with the model group, the QKF and donepezil groups showed significantly elevated cellular viability (*P* < 0.01). The 25 mg mL^−1^ QKF group showed an obviously improved effect compared to the QKF groups treated with the other tested doses (*P* < 0.01), indicating the concentration-dependent neuroprotective effect of QKF.

### 3.6. Inhibiting or Activating p38 MAPK Affected the Hippocampal Neuron Morphology

**Figure 6:**
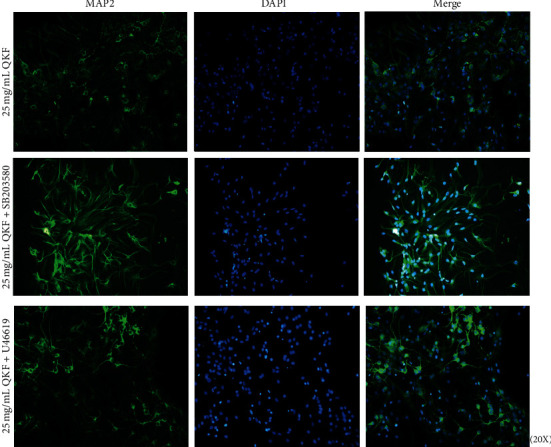
Immunofluorescence images of microtubule-associated protein-2 (green) and nucleus (blue) in the QKF group, QKF + SB203580 group, and QKF + *U*46619 group (original magnification, ×200). QKF, Qingxin kaiqiao fang.

The abovementioned results demonstrated that 25 mg mL^−1^ QKF showed the best effect in terms of preserving the morphology and inhibiting the apoptosis of hippocampal neuronal cells among the three doses of QKF assessed. Therefore, 25 mg mL^−1^ QKF was used for the subsequent experiments. In addition, p38 MAPK activity was inhibited by SB203580 and activated by U46619 to determine whether the antiapoptotic effect of QKF on neurons was mediated by p38 MAPK silencing. Neurons in the 25 mg mL^−1^ QKF + SB203580 group were plumper, their synapses were longer and thicker, and the nerve fiber network was much more elaborate and dense compared to that in the other two groups (Figure 6).

### 3.7. Inhibiting or Activating p38 MAPK Affected Hippocampal Neuronal Apoptosis

**Figure 7:**
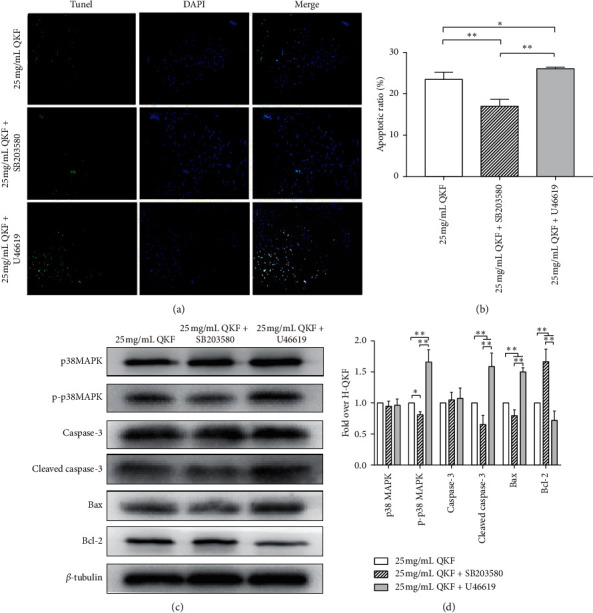
25 mg mL^−1^ QKF alleviates neuronal apoptosis by p38 MAPK silencing. (a) TUNEL staining in each group of hippocampal neurons (original magnification, ×200). (b) Statistical analysis results of TUNEL staining. (c) The gray value of associated proteins in each group. (d) Relative protein expression in each group, as detected by WB analysis. Data are expressed as mean ± SD (*n* = 5); significant differences between the two groups are indicated as ^*∗*^*P* < 0.05 and ^*∗∗*^*P* < 0.01. QKF, Qingxin kaiqiao fang.

As shown in Figures 7(a) and 7(b), the 25 mg mL^−1^ QKF + SB203580 group showed a lower apoptotic rate than the 25 mg mL^−1^ QKF group (*P* < 0.01), and the rate of hippocampal neuron apoptosis was higher in the 25 mg mL^−1^ QKF + *U*46619 group than that in the 25 mg mL^−1^ QKF group (*P* < 0.05). The apoptotic rate in the SB203580-treated group was notably lower than that in the 25 mg mL^−1^ QKF + *U*46619 group.

WB analysis was employed to measure the extent of p38 MAPK phosphorylation and the protein expression levels of p38 MAPK, caspase-3, cleaved caspase-3, Bax, and Bcl-2 among the three groups. As shown in Figures 7(c) and 7(d), compared with the 25 mg mL^−1^ QKF group, the SB203580-treated group showed higher expression levels of Bcl-2 (*P* < 0.01) and lower expression levels of p-p38 MAPK, cleaved caspase-3, and Bax (*P* < 0.05, *P* < 0.01). The expression levels of p-p38 MAPK, Bax, and cleaved caspase-3 in the U46619-treated group were significantly higher than those in the 25 mg mL^−1^ QKF group (*P* < 0.01), and the level of Bcl-2 was markedly lower than that in the 25 mg mL^−1^ QKF group (*P* < 0.01). There was no evident difference in the levels of p38 MAPK and caspase-3 (*P* > 0.05) in the 25 mg mL^−1^ QKF + SB203580 or 25 mg. mL^−1^ QKF + *U*46619 groups compared with the H-QKF group. These results demonstrated that inhibiting p38 MAPK blocked apoptosis, while p38 MAPK activation produced an inverse effect compared to that of p38 MAPK pathway inhibition.

### 3.8. Inhibiting or Activating p38 MAPK Affected Cell Viability

**Figure 8:**
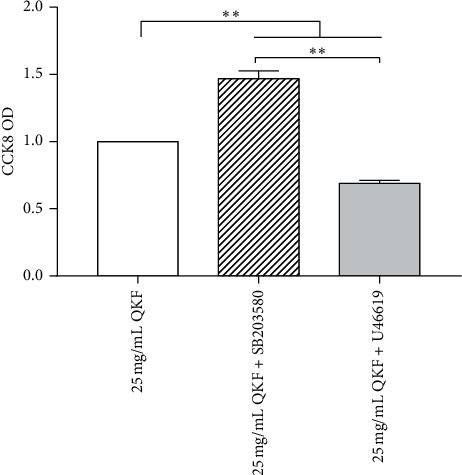
25 mg mL^−1^ QKF promotes cell viability by p38 MAPK silencing. Data are expressed as mean ± SD (*n* = 5); significant differences between the two groups are indicated as ^*∗*^*P* < 0.05 and ^*∗∗*^*P* < 0.01. QKF, Qingxin kaiqiao fang.

The cell survival rate was calculated as (OD _experimental group_−OD _blank group_)/(OD _25 mg/mL__QKF group_ - OD _blank group_) × 100%. In comparison with that in the 25 mg mL^−1^ QKF group, hippocampal neuron viability in the 25 mg mL^−1^ QKF + SB203580 group was obviously increased (*P* < 0.01), and cell viability in the 25 mg mL^−1^ QKF + *U*46619 group was notably decreased (*P* < 0.01) (Figure 8). These findings indicated that inhibiting p38 MAPK activity elevates hippocampal neuron viability and that activating the p38 MAPK pathway inhibits hippocampal neuron viability.

## 4. Discussion

A*β*_25-35_, a short fragment of the A*β* peptide that is related to AD, has been found in experimental studies to have amyloidogenic, aggregative, and neurotoxic features. A*β*_25-35_ sedimented faster, indicating an increased tendency toward aggregation compared with A*β*_1-42_, which is another peptide widely used to develop AD models in vivo and in vitro and to generate apoptotic signals leading to cell death [14, 15]. Neuronal apoptosis, which is an important cell process and the main mechanism of neuronal death, plays an essential role in neurodegeneration in AD. The significance of apoptosis in the pathogenesis of AD has attracted increasing attention [16]. In a normal physiological environment, apoptosis and the natural antiapoptotic defense system exist in a dynamic balance and the excessive accumulation and deposition of A*β* disrupt this balance [17]. The present study revealed that A*β*25-35 at 10 *μ*mol·L-1 destroyed cell morphology, disrupted the connections between neurons, decreased cell viability, and increased the number of TUNEL-positive cells. However, these effects were improved after treatment with QKF, which has been used to treat AD for many years and has shown remarkable effects on early symptoms such as cognitive dysfunction and behavioral symptoms [18].

**Figure 9:**
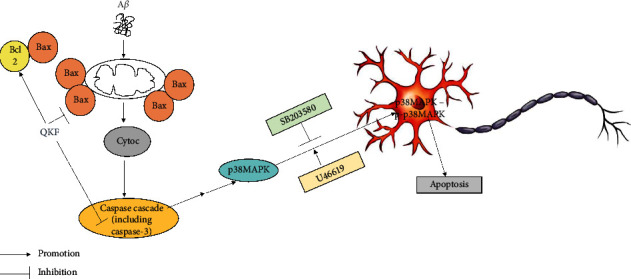
A*β* treatment significantly overexpressed Bax protein and has a little effect on reducing the expression of Bcl2 protein. These changes released cytochrome c from mitochondria, which resulted in activation of the caspase cascade, including caspase-3, the downstream protein of the caspase family. The activation of caspase cascade indirectly stimulated p38 MAPK, resulting in p38 MAPK phosphorylation. Finally, p-p38 MAPK led to apoptosis. QKF enhanced the expression of Bcl-2 and reduced the expression of Bax and cleaved caspase-3, thus affecting subsequent steps.

Network pharmacology prediction was applied to deeply explore the potential mechanisms underlying the effects of QKF on AD. We first screened the active compounds of QKF and identified the target proteins of QKF. Then, the QKF-compound-target-AD network was constructed, and enrichment analysis was performed. Our analysis indicated that QKF was mainly targeted in the cell cycle, cell proliferation, apoptosis, and inflammation in the treatment of AD. It can be seen from the above enrichment analysis that both the MAPK pathway and the apoptotic process are very important. In our previous in vivo study, QKF was found to successfully ameliorate memory impairment and inhibit apoptosis in APP/PS1 double transgenic mice through its effects on the p38 MAPK pathway [19]. Mammalian p38 MAPK, a member of the MAPK family, is mainly involved in promoting apoptosis. A*β* aggregated as extracellular plaques phosphorylates p38 MAPK, which then induces tau hyperphosphorylation, reduces synaptic plasticity, and eventually induces neuronal apoptosis [20]. The results of the WB analysis in our present in vitro study showed that the phosphorylation of p38 MAPK was significantly increased in the model group compared with the control group. In the QKF-treated group, a significant decrease in the protein expression of p-p38 MAPK was observed compared with the model group. In addition to the role of p38 MAPK in apoptosis, a family of intracellular cysteine proteases known as caspases are known to exhibit apoptotic effects and to be involved in the intrinsic mitochondrial apoptosis pathway [21]. Caspase-3, the final executor of apoptosis and the best-characterized apoptosis executor within the caspase family, can be cleaved by an activated upstream caspase [22]. Following the exposure of cells to apoptotic stimuli, caspase activation has been demonstrated to rely on the presence of cytochrome c released from mitochondria; however, apoptosis can be prevented by the presence of Bcl-2 in these organelles [23]. Bcl-2 is an integral membrane protein located mainly on the outer membrane of mitochondria that are known for improving cell survival and preventing cells from undergoing apoptosis [24]. In contrast, the related protein Bax facilitates apoptosis and counters the antiapoptotic role of Bcl-2^9^ (the potential mechanism is shown in Figure 9). The results of the WB analysis in the present study showed that QKF, especially at a dose of 25 mg mL^−1^, rescued hippocampal neurons from A*β*-induced apoptosis by reducing the expression of Bax and cleaved caspase-3 and by enhancing the expression of Bcl-2.

Because p38 MAPK plays a role in the process of apoptosis, the p38 MAPK inhibitor SB203580 has been used to decrease p38 MAPK activation by many researchers [25, 26]. In this study, to verify that the inhibition of p38 MAPK activity has an antiapoptotic effect, U46619, an agonist of p38 MAPK, was applied [27, 28]. Our results demonstrated that the 25 mg mL^−1^ QKF + SB203580 group, but not the U46619-treated group, exhibited increased preservation of neuronal morphology, enhanced neuron viability, a decreased number of TUNEL-positive cells, upregulated expression of an antiapoptotic protein, and downregulated expression of proapoptotic proteins. These findings suggest that QKF prevents apoptosis in AD by silencing the p38 MAPK pathway.

## 5. Conclusion

QKF, especially the high-dose group of 25 mg mL^−1^, may inhibit A*β*_25-35_-induced hippocampal neuron apoptosis by blocking the p38 MAPK pathway. Moreover, our ﬁnding also demonstrated that network pharmacology was a reliable way to ﬁnd targets and possible mechanisms of traditional Chinese medicine.

## Data Availability

The basic studying data used to support the findings of this study were supplied by Dr. Tian-Qi Wang under license and so cannot be made freely available. Requests for access to these data should be made to Dr. Tian-Qi Wang, tenkiou@foxmail.com.

## Supplementary Materials

Supplementary Materials The data of active ingredients and targets are listed in Supplementary Tables 1, 2, 3, and 4. The data of PPI Network and Functional Enrichment Analysis can be seen in Supplementary Tables 5, 6, 7, 8, and 9.
